# Serum-based inhibition of pitviper venom by eastern indigo snakes (*Drymarchon couperi*)

**DOI:** 10.1242/bio.040964

**Published:** 2019-03-01

**Authors:** Scott M. Goetz, Sara Piccolomini, Michelle Hoffman, James Bogan, Matthew L. Holding, Mary T. Mendonça, David A. Steen

**Affiliations:** 1 Department of Biological Sciences, Auburn University, Auburn, AL 36849, USA; 2 Orianne Center for Indigo Conservation, Central Florida Zoo & Botanical Gardens, Eustis, FL 32736, USA; 3 Department of Biological Sciences, Florida State University, Tallahassee, FL 32304, USA; 4 Georgia Sea Turtle Center, Jekyll Island Authority, Jekyll Island, GA 31527, USA

**Keywords:** *Agkistrodon*, Antagonistic interactions, Hemolytic, Reptile, Snake venom metalloproteinases

## Abstract

When organisms possess chemical defenses, their predators may eventually evolve resistance to their toxins. Eastern indigo snakes (*Drymarchon couperi*; EIS) prey on pitvipers and are suspected to possess physiological resistance to their venom. In this study, we formally investigated this hypothesis using microassays that measured the ability of EIS blood sera to inhibit (A) hemolytic and (B) snake venom metalloproteinase (SVMP) activity of copperhead (*Agkistrodon contortrix*) venom. To serve as controls, we also tested the inhibitory ability of sera from house mice (*Mus musculus*) and checkered gartersnakes (*Thamnophis marcianus*), a snake that does not feed on pitvipers. Sera from both EIS and gartersnakes inhibited over 60% of SVMP activity, while only EIS sera also inhibited venom hemolytic activity (78%). Our results demonstrate that EIS serum is indeed capable of inhibiting two of the primary classes of toxins found in copperhead venom, providing the first empirical evidence suggesting that EIS possess physiological resistance to venom upon injection. Because we documented resistance to hemolytic components of pitviper venom within EIS but not gartersnakes, we speculate this resistance may be driven by selection from feeding on pitvipers while resistance to SVMP may be relatively widespread among snakes.

## Abstract (precis)

**Summary:** We confirm eastern indigo snakes possess a physiological resistance to pitviper venom and speculate that venom resistance in snakes may be a product of antagonistic interactions and phylogenetically conserved traits.

## INTRODUCTION

Physiological resistance to toxins may evolve in predators that eat chemically-defended prey (Brodie, 1990; Rowe and Rowe, 2008) and selection for greater resistance is predicted to be stronger in predators that exhibit greater diet specialization (Arbuckle et al., 2017). Animal poisons and especially venoms are complex mixtures of toxins (Casewell et al., 2013; Fox and Serrano, 2008; Fry et al., 2009, 2012). The amount of damage caused by specific toxins, however, varies greatly and resistance may be achieved by inhibition of relatively few toxins (Arbuckle et al., 2017).

Blood sera components are one potential mechanism facilitating venom resistance, as they may bind to venom toxins and neutralize them, thereby inhibiting venom activity and minimizing damage. This serum-based toxin resistance appears to have independently evolved in a taxonomically-diverse suite of organisms in response to different ecological pressures (Arbuckle et al., 2017; Holding et al., 2016a; Perez et al., 1978). For example, resistance to pitviper venom has been documented in both snake prey and predators (de Wit, 1982; Perez et al., 1979; Pomento et al., 2016; Poran et al., 1987; Voss and Jansa, 2012), including three snake species that eat venomous prey (Lomonte et al., 1982; Tomihara et al., 1988; Weinstein et al., 1992) as well as pitvipers themselves, presumably as a form of autoresistance (Clark and Voris, 1969; Weinstein et al., 1991). Perhaps the best studied example of serum-based resistance to pitviper venom involves California ground squirrels (*Spermophilus beecheyi*) inhibiting the activity of rattlesnake (*Crotalus* spp.) venom. Ground squirrels are a major dietary component of many co-occurring rattlesnakes and ground squirrel sera contains factors that neutralize the digestive and hemostatic effects of pitviper venom (Biardi et al., 2006). Furthermore, detailed investigations have revealed among-population variation in both snake venom activity and squirrel resistance that suggests a co-evolutionary relationship (Biardi et al., 2006; Holding et al., 2016b) and supports the idea that prey capture, not antipredator defense, is likely the primary selective factor acting on snake venom evolution (Fry et al., 2008; Li et al., 2005; Richards et al., 2012).

Eastern indigo snakes (*Drymarchon couperi*; EIS) are predators of a variety of venomous snakes, and thus provide an appropriate model organism to explore ideas related to the evolution of venom resistance. Historically restricted to southern portions of the southeastern coastal plain of USA, EIS are associated with open-canopy pine savannahs and are considered dietary generalists preying on a variety of mammals, birds, reptiles, and amphibians. Prey records indicate that snakes, including a number of pitviper species, are the most commonly consumed food item (Steen et al., 2016; Stevenson et al., 2010). An experimental investigation of EIS response to prey odors revealed a preference for pitvipers over all other prey scents tested (Goetz et al., 2018). Together, qualitative and quantitative evidence indicates that EIS and pitvipers likely share a co-evolutionary history shaped by predator/prey dynamics.

Long-standing suggestions that snakes in the genus *Drymarchon* are resistant to the effects of pitviper venom (Boos, 2001; Keegan and Andrews, 1942; Mole, 1924) primarily stem from observations of successful predation of pitvipers by indigo snakes. Survival following possible envenomation, however, serves as a poor test of resistance (Arbuckle et al., 2017). For example, pitvipers can meter the quantity of venom injected during bites (Hayes, 2008; Hayes et al., 2002) thus it is not possible to estimate the amount of venom, if any, delivered during an observed bite. Moreover, envenomation by pitvipers requires penetration of outer epithelial layers (i.e. wound formation) and the large, thick scalation of EIS likely serves as formidable barrier to penetration of snake fangs. Finally, the predatory sequence of EIS typically begins by grasping and crushing the head of snake prey; therefore, toxic defenses may be bypassed altogether by subduing pitvipers before they can strike (Keegan and Andrews, 1942; Moulis, 1976). A more direct, experimental approach is necessary to determine if EIS possess physiological mechanisms to inhibit venom protein activity.

Here, we used a pair of venom activity assays to formally evaluate the ability of EIS blood sera to inhibit two of the primary groups of toxins in copperhead venom (*Agkistrodon contortrix*; Linnaeus, 1766). We first assessed serum inhibition of hemolytic factors, including toxins that damage erythrocytes and disrupt hemostasis (Biardi and Coss, 2011). We also investigated the inhibition of snake venom metalloproteinases (SVMPs) that damage proteins in the extracellular matrix and hydrolyze collagen (Biardi et al., 2011; Holding et al., 2016b; Pomento et al., 2016). Because collagen is found in both the lining of blood vessels and muscle tissue, SVMPs can cause hemorrhagic effects.

Our objective was to evaluate the hypothesis that EIS possess serum-based resistance to pitviper venom; we assumed a strong signal of venom inhibition would suggest the presence of circulating inhibitor molecules in the blood. We predicted EIS serum would exhibit greater toxin inhibition compared with house mouse (*Mus musculus*) serum, which lacks venom protein inhibitors and served as our experimental control. To add additional ecological context to our investigation, we also evaluated the inhibitory ability of serum from checkered gartersnakes (*Thamnophis marcianus*; Baird and Girard, 1853) that do not prey on pitvipers (Ernst and Ernst, 2003). We predicted greater inhibition by EIS compared with gartersnakes because the former is more likely to participate in antagonistic interactions with pitvipers.

## RESULTS

**Fig. 1.**
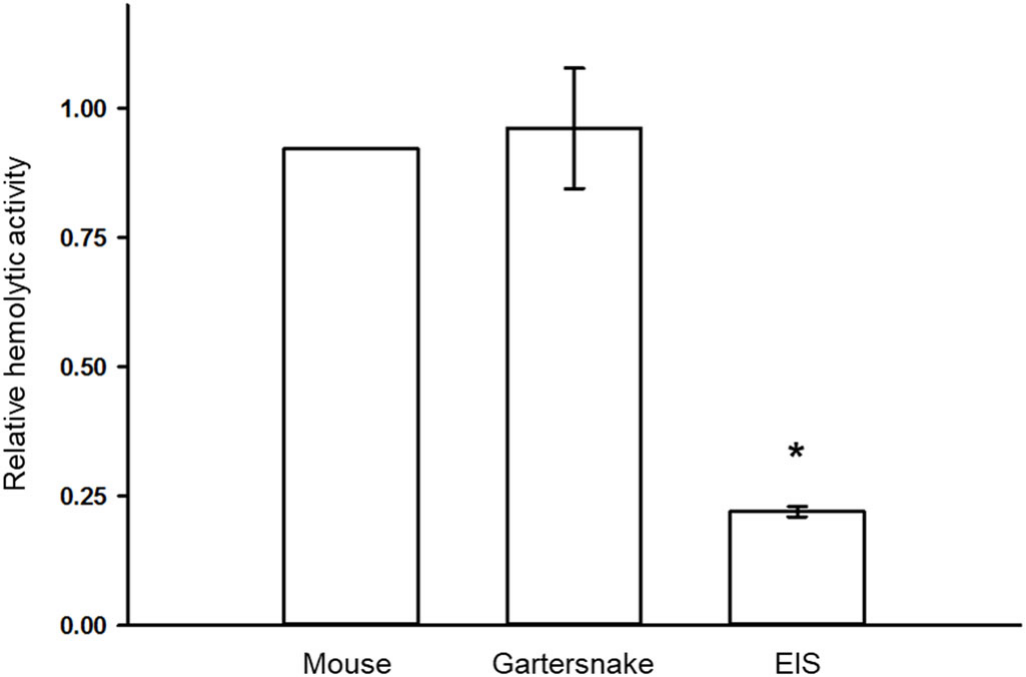
**Hemolytic activity (mean±s.e.m.) of a pooled sample of copperhead (*A. contortrix*) venom incubated with blood sera from either mice (*M. Musculus*; *n*=1, pooled), eastern indigo snakes (*D. couperi*, EIS; *n*=15), or checkered gartersnakes (*T. marcianus*; *n*=6).** Activity is expressed relative to a venom-only control and asterisk indicates a significant difference (*P*<0.05) of sera-incubated treatments. Hemolytic activity of venom was reduced following incubation with EIS sera (*P*<0.001) but not mouse sera (*P*=0.716) or gartersnake sera (*P*=0.811). Venom-only control was replicated nine times, venom+sera treatments were performed in triplicate for each individual subject and the average was used as the unit of analysis for statistical comparisons. Significance was analyzed by fitting data to a linear regression model.

Serum inhibition of venom hemolytic activity varied among the species studied. The linear regression model revealed a significant effect of serum origin on inhibition (*F*_3,19_=42.96, *P*<0.001, Fig. 1). In comparison to the hypothesized mean, incubation of venom with sera from EIS reduced the hemolytic activity of copperhead venom (*P*<0.001) by an average of 78% (range=68–83%). On the contrary, hemolytic activity of venom was not inhibited following incubation with mouse sera (*P*=0.716) or gartersnake sera (*P*=0.811). Furthermore, there was no difference in the inhibitory ability between mouse and gartersnake sera (*P*=0.813). Significant inhibition of venom hemolytic activity by EIS, but not by gartersnake or mouse sera, is consistent with our major prediction and thus supports the hypothesis that EIS possess serum-based resistance to pitviper venom.

**Fig. 2.**
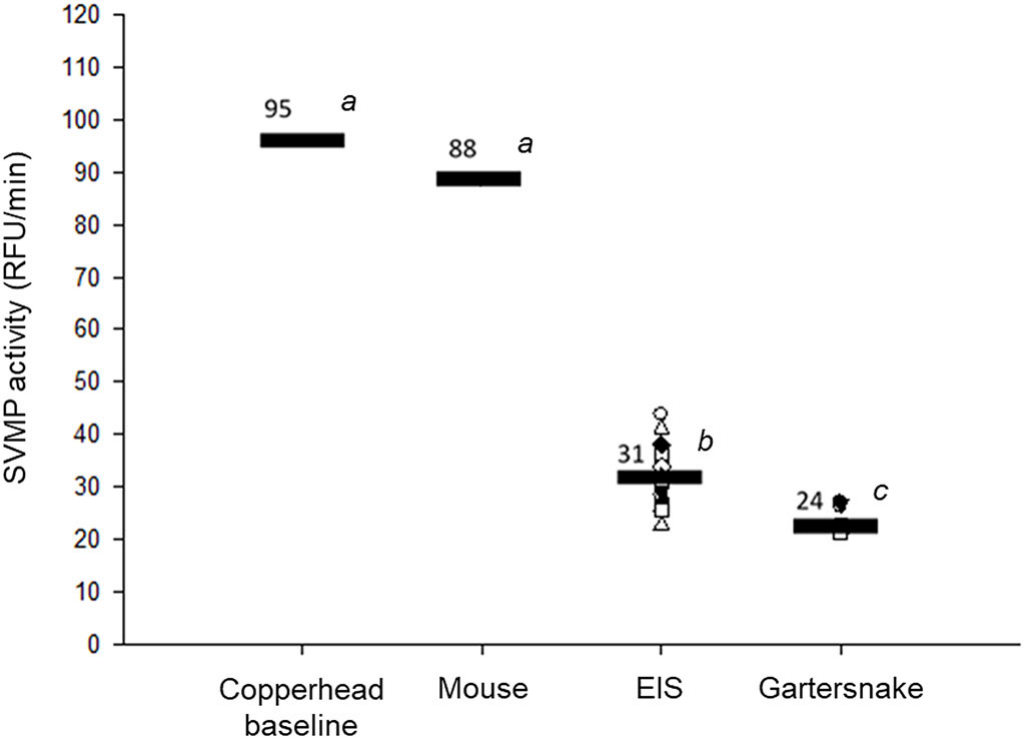
**SVMP activity of a pooled sample of copperhead (*A. contortrix*) venom alone, and incubated with sera from either mice (*M. musculus*; *n*=1, pooled), eastern indigo snakes (*D. couperi*, EIS; *n*=18), or checkered gartersnakes (*T. marcianus*; *n*=6).** SVMP activity is represented as a rate of relative fluorescence units (RFU_520nm_ min^−1^). Sera samples from individual subjects were replicated in triplicate and the average was used as the unit of analysis for statistical comparisons. Horizontal black bars represent group means, denoted by the number above the bar, and corresponding letters indicate significantly different (*P*<0.05) groupings. Vertically-aligned symbols are the mean values of individual subjects. SVMP activity of venom was reduced following incubation with sera from EIS (*P*<0.001) and gartersnakes (*P*<0.001) but not mice (*P*=0.689). Gartersnake sera was more effective than EIS sera at inhibiting SVMP activity (*P*=0.012). Significance was analyzed by fitting data to a linear regression model.

Both EIS and gartersnakes inhibited the SVMP activity of copperhead venom. The linear regression model revealed a significant impact of serum origin on venom inhibition (*F*_3,22_=70.23, *P*<0.001, Fig. 2). This effect was characterized by a lack of inhibition during incubation with mouse serum proteins (*P*=0.689) but a significant reduction of SVMP activity following incubation with EIS sera (*P*<0.001) and gartersnake sera (*P*<0.001). EIS sera caused a >66% reduction in SVMP activity. Similarly, SVMP activity was reduced by >74% by incubation with gartersnake sera. There was also a difference in the inhibitory ability between the two snake sera treatments such that gartersnake sera was more effective than EIS sera at inhibiting SVMP activity (*P*=0.012). These results are aligned with the prediction that EIS should inhibit copperhead SVMP activity more effectively than mice, while the effectiveness of gartersnake serum at SVMP inhibition was unexpected and suggests the possibility of partial venom resistance in gartersnakes.

## DISCUSSION

Exploring physiological responses to venom is crucial to understanding how venom resistance evolves, the mechanisms by which it functions, and whether the same or different physiological solutions are employed to deal with envenomation. Our investigation of serum-based resistance to pitviper venom by EIS provides evidence that EIS, as predators of pitvipers, have evolved venom resistance as a trophic adaptation. Specifically, we provide *in vitro* functional evidence that EIS possess a physiological resistance to both hemolytic and SVMP activities of copperhead venom. Venom resistance in EIS fits the conceptual model that antagonistic interactions can drive the evolution of resistance (Arbuckle et al., 2017; Brodie and Brodie, 1999; Holding et al., 2016a). The ability of gartersnakes to inhibit SVMPs was unexpected and we speculate that it may represent a phylogenetically conserved trait because gartersnakes have limited ecologically-relevant interactions with pitvipers.

Our finding that EIS did not completely inhibit either type of venom toxin tested is consistent with observations of EIS following purported envenomation by pitvipers. For example, pitviper bites rarely appear to be fatal to indigo snakes (*Drymarchon* spp.), but have been noted to induce localized swelling and skin necrosis (Moulis, 1976; Boos, 2001). Likely the severity of complications arising from envenomation in EIS is dependent on the amount of venom injected, the location of the bite, and the potential for variation in physiological resistance among individual EIS. Known serum-based venom inhibitors in mammals and reptiles are hypothesized to titrate the venom out of the bitten animal's body via irreversible binding and inactivation (reviewed in Holding et al., 2016a), and thus the relative concentrations of venom and venom inhibitors will impact symptom severity, particularly near the bite site where venom concentration is initially very high. Future work detailing EIS responses to variations of the amount and type of venom are necessary to accurately characterize survival thresholds.

EIS occupy a similar ecological niche as snakes of the genus *Lampropeltis* (kingsnakes) that also possess serum-based inhibition of pitviper venom (Rosenfeld and Glass, 1940; Bonnett and Guttman, 1971; Philpot et al., 1978; Weinstein et al., 1992). Both kingsnakes and EIS are ophiophagous and exhibit a preference for pitviper prey (Weldon and Schell, 1984; Goetz et al., 2018) but are not considered dietary specialists. For predators, theory suggests trophic dietary specialization is the greatest primary selective pressure on the evolution and efficiency of toxin resistance; however, additional ecological inequalities may also drive selective pressures (Arbuckle et al., 2017). For example, the cost of maintaining resistance may be reduced for predators of pitvipers if selection on venom is prey-mediated. This suggestion is supported by unique defensive postures exhibited by pitvipers in response to ophiophagous snakes, including EIS (reviewed in Weldon et al., 1992). In addition to defensively striking at or biting approaching snake predators, pitvipers often exhibit a ‘body-bridging’ posture in which they raise their body in a forward, vertical loop, presumably hiding their head or making it difficult to grasp (Klauber, 1927; Carpenter and Gillingham, 1975). The adoption of specific behavioral defenses in response to snake predators suggest that venom alone may be an insufficient defense and that pitviper prey, as opposed to predators, may exert stronger selection pressure on venom.

It is possible that inhibition of venom hemolytic activity in EIS is not associated with a serum protein, but instead by high vitamin E concentrations consistently found in EIS serum: as speculated by Dierenfeld et al. (2015). Hemolytic activity of viper venoms is negatively associated with vitamin E concentrations in human subjects (Mukherjee et al., 1998). Thus, it is possible that the high vitamin E concentrations of EIS are a direct physiological adaptation to resisting pitviper hemolytic toxins, and that vitamin E comprises all or part of EIS serum's ability to inhibit venom hemolytic activity. If this were so, it would represent a novel form of serum resistance compared to the protein inhibitors found in the serum of other resistant taxa (Domont et al., 1991; Dunn and Broady, 2001; Thwin and Gopalakrishnakone, 1998). Alternatively, high vitamin E concentrations could be a non-adaptive byproduct of EIS diet that happen to confer an advantage. If gartersnakes lack elevated vitamin E, this could explain why they only possess resistance to SVMP activity.

Gartersnake sera inhibited SVMP activity but not hemolytic proteins and this may explain why previous studies investigating resistance to pitviper venom by gartersnakes (*Thamnophis* spp.) reported conflicting results. For example, Weinstein et al. (1992) suggest that gartersnakes exhibit no neutralization capacity for pitviper venom based on low survivorship of mice injected with venom previously incubated with gartersnake sera. In contrast, earlier studies that directly injected pitviper venom into gartersnakes reported high survivorship (Keegan and Andrews, 1942; Swanson, 1946). These studies used survival as the sole measure of resistance and did not explore inhibition of specific venom toxins. Thus, inhibition of SVMP by gartersnakes may be sufficient to facilitate survival in some, but not all, instances. It is unclear, however, what the ecological significance of partial venom resistance is for gartersnakes. Serum inhibition of SVMP could be a response to predation pressure: both copperheads and closely-related cottonmouths (*Agkistrodon piscivorus*) are known, at least occasionally, to prey on gartersnakes (Ernst and Ernst, 2003). Alternatively, resistance to SVMP may represent a phylogenetically conserved trait present in many snakes; future phylogenetic studies incorporating a diversity of species may provide clarity on this question.

We have confirmed what has been long-suspected for EIS, that their blood sera is able to inhibit pitviper venom, suggesting an evolutionary response to at least some venom components that allow the safe consumption of venomous snakes. Given the dietary habits and prey preferences of EIS (Steen et al., 2016; Stevenson et al., 2010; Goetz et al., 2018) and the fact that SVMPs and hemolytic proteins are key components of most of the venoms of pitvipers within the geographic distribution of EIS, our results lead us to speculate that EIS are also resistant to venom from other co-occurring pitviper species. We further speculate that the evolution of resistance to hemolytic toxins in EIS is an adaptation for predation, possibly indicating a co-evolutionary relationship while the origin of SVMP inhibition remains unclear. Finally, we suggest the growing number of species capable of inhibiting pitviper venom warrant further study into the complex ecological and evolutionary processes driving the evolution of venom resistance as well as possible reciprocal co-evolutionary responses by pitvipers.

## MATERIALS AND METHODS

### Sample collection

Blood samples were collected from snakes via puncture of the ventral coccygeal vein using a 22-gauge needle and placed in a 5 ml test tube topped with paraffin. Blood samples were initially stored on ice and subsequently in a refrigerator (∼3°C) overnight to allow the blood to clot. Following removal of blood clots, serum samples were centrifuged for 10 min at 2000 ***g*** and placed in a −80°C freezer for long-term storage. A pooled sample of mouse (*M. musculus*) sera was commercially purchased (Sigma-Aldrich, cat. M5905) and reconstituted in phosphate buffered solution (PBS).

We collected EIS blood samples from captive individuals at the Orianne Center for Indigo Conservation (OCIC) in Eustis, USA. EIS were between 2 and 7 years old with a mean mass of 1515±86.5 g (±s.e.m.; range=1049–2411 g). All individuals were hatched in captivity from 14 egg clutches produced by the pairing of wild-caught adults collected from Georgia and Florida, USA as well as three adults of unknown origin. Snakes were singly housed in either indoor plastic enclosures or in open-air outdoor enclosures. We collected checkered gartersnake blood samples from captive individuals singly housed in a rack system at Auburn University, USA. Gartersnakes were from two litters and all were under 1 year of age at the time of sample collection. Gartersnakes were the product of a multigenerational captive breeding program: the geographic source of parental snakes is unknown but believed to be Texas. Both EIS and gartersnakes lacked any past exposure to pitviper snakes.

Lyophilized copperhead venom was commercially purchased (Texas A&M National Natural Toxins Research Center, Kingsville, TX, USA). The venom was pooled from three individual adult copperheads of unknown sex that were wild-caught in Harris County, TX, USA. Following the methods of Biardi et al. (2011), venom was reconstituted in buffer containing 50 mM Tris–HCl, 5 mM CaCl_2_, and 0.05% Brij, pH 7.6 at a concentration of 50 mg ml^−1^ for storage at −20°C.

### Serum-based inhibition of hemolytic toxins

In this experiment, we investigated the ability of blood sera from EIS (*n*=15), gartersnakes (*n*=6) and mice (*n*=1, pooled) to inhibit hemolytic toxins in copperhead venom following the methods of Biardi and Coss (2011). To start, we punched 3 mm diameter wells into BBL stacker plates (BD Life Sciences, Franklin Lakes, NJ, USA, cat. 221165) containing 5% defibrinated sheep's blood in Columbian agar. Venom was diluted to a concentration of 10 mg ml^−1^. All wells were filled with a total volume of 20 µl. To determine baseline hemolysis activity of venom, wells were filled with a mixture of 10 µl of venom and 10 µl of buffer, but no serum. Treatment wells were filled with a mixture of 10 µl of venom, 5 µl of buffer and 5 µl of serum. We also prepared buffer-only and serum+buffer controls. After filling wells, plates were sealed with parafilm and incubated at 37°C for 48 h. Then, to quantify the hemolytic activity of venom, we photographed plates and two independent observers, blind to treatment assignments, scored each well by calculating the area of the hemolytic zone (i.e. area of transparent agar) surrounding wells by measuring two perpendicular diameters using ImageJ software (1.x; Schneider et al., 2012). The average of the scores recorded by the two observers was used for data analysis.

Venom+buffer, serum+buffer and buffer-only control treatments were replicated nine times and venom+serum treatments were performed in triplicate. All replicate wells were located on different agar plates and their average was used as the unit of analysis for statistical comparisons. The average area of hemolysis surrounding wells containing only buffer was subtracted from venom-only replicates; similarly, the area of hemolysis resulting from serum+buffer were subtracted from venom+serum treatments.

### Serum-based inhibition of snake venom metalloproteinases

In this experiment, we investigated the ability of blood sera from EIS (*n*=18), gartersnakes (*n*=6), and mouse sera (*n*=1, pooled), to inhibit SVMP activity of copperhead venom following the methods of Biardi et al. (2011). The enzymatic activity of copperhead venom was quantified using DQ fluorogenic gelatin (Thermo Fisher Scientific, cat. 12054) that emits fluorescence during proteolytic digestion and served as a model of collagen tissue and extracellular matrix. Gelatin was diluted to a concentration of 0.02 mg ml^−1^ in a buffer containing 50 mM Tris-HCL, 150 mM NaCl, 5 mM CaCl_2_, 0.2 mM NaN_3_, pH 7.6. Venom was diluted to a concentration of 0.05 mg ml^−1^ in a buffer containing 50 mM Tris-HCL, 5 mM CaCl_2_, 0.05% Brij, pH 7.6.

The microassay was conducted in black, flat-bottom 96-well plates (Corning, NY, USA) at 25°C. We determined the hypothesized baseline SVMP activity of the venom by measuring fluorescence of a mixture containing 50 µl of gelatin, 100 µl of venom and 50 µl of PBS. To test the ability of serum to inhibit SVMPs, we first determined the protein concentration of all serum samples using a Bradford Method Protein Assay (VWR, Randor, PA, USA, cat. 97064-924) and subsequently standardized samples by dilution in PBS to a final concentration of 10 mg ml^−1^. Then 5 µl of diluted serum from each sample was mixed with 45 µl of PBS. The 50 µl serum+buffer samples were incubated with 100 µl of venom for 30 min at 23°C before adding 50 µl of gelatin and measuring fluorescence. We also prepared controls containing buffer and either only gelatin, venom or serum.

Less than 2 min after combining gelatin and venom or venom+serum mixtures, fluorescence was measured once every min for 45 min in a microplate reader (BioTek, Cytation 3 Imaging Reader). Fluorescence was set to 520 nm emission and 460 nm excitation wavelengths and all replicates were measured simultaneously on the same plate. Background fluorescence of controls was subtracted and SVMP activity was expressed as the change in relative fluorescence units (RFU_520nm_) over time. We determined the linear part of the reaction (2–8 min) and used these data to calculate the slope (RFU min^−1^) which we used as our measure of maximum SVMP activity in each reaction well. All treatments, including controls, were replicated in triplicate and the average was used as the unit of analysis for statistical comparisons.

### Statistical analysis

For the hemolytic toxin assay, data was fit to a linear regression model comparing treatments containing sera from EIS, gartersnakes, and mice to the venom-only treatment that represents the hypothesized mean for baseline hemolytic activity of copperhead venom. For investigation of SVMP inhibition, we fit the reaction slopes for each sample to linear regression model comparing sera from EIS, gartersnakes, and mice, to baseline copperhead venom activity (95.7 RFU_520nm_ min^−1^) as the hypothesized mean. The linear model was re-leveled to change reference categories allowing pairwise comparisons of treatments. All statistical analyses were completed using R version 3.2.4 (R Core Team, 2016). Our threshold for statistical significance was *P*<0.05.
